# Plan ahead, or wing it? How storm-petrel parents adjust food delivery to young chicks

**DOI:** 10.1093/beheco/araf127

**Published:** 2025-11-07

**Authors:** Robert A Mauck, Liam U Taylor, Samuel C Neirink, Kayla E Lichtner, Sarah E Chapman, James H Veitch, Ian T Kyle, Mark F Haussmann, Patricia L Jones

**Affiliations:** Kenyon College, Gambier, OH 43022, USA; Bowdoin College, Brunswick, ME 04011, USA; Bowdoin College, Brunswick, ME 04011, USA; Bucknell University, Lewisburg, PA 17837, USA; Bucknell University, Lewisburg, PA 17837, USA; Neeto Industries, Brisbane 4306, Australia; Bowdoin College, Brunswick, ME 04011, USA; Bucknell University, Lewisburg, PA 17837, USA; Bowdoin College, Brunswick, ME 04011, USA

**Keywords:** allocation, Burrow Scale Monitor, chick age, food delivery, foraging, parental investment

## Abstract

Parents must decide how to allocate energy gained from foraging between self and offspring. Storm-petrels (Procellariiformes: Hydrobatidae) are pelagic seabirds that travel hundreds of kilometers across multiple days before returning to the nesting burrow to feed a dependent chick. Parents return to the nest with food stored in the proventriculus, a portion of which is regurgitated to their offspring. As the chick grows, provisioning demands increase. However, it is unknown whether parents meet this increasing demand by (1) altering their foraging strategies to acquire more food or (2) allocating a greater proportion of their intake to the chick. We designed, validated, and implemented a new technology—the Burrow Scale Monitor—to measure Leach's storm-petrels (*Hydrobates leucorhous*) as they entered and exited the nesting burrow. We monitored breeding adults over the first 30 d of chick rearing to determine whether storm-petrel parents adjust their foraging intake to the age of the chick or simply adjust energy allocation at the nest. Food delivery increased with chick age, but this increase was driven to a much greater extent by parents delivering a greater proportion of their body mass as food (ie, a shift in parental allocation) rather than by adults adjusting their foraging strategy to match chick age. Only by measuring adult body mass on arrival and exit at the nesting burrow could we understand how parents adapt their provisioning strategy to the increasing demands of the growing chick.

## Teaser

New technology reveals how storm-petrels choose to allocate more food to their single chick as it grows older. These pelagic seabirds fly hundreds of kilometers to forage, returning to their underground nests only at night. We developed the Burrow Scale Monitor to automatically weigh wild storm-petrels as they enter and exit their nesting burrows. Our results highlight the dynamic energetic decisions parents make as chicks grow, while our open-source methods offer new possibilities for studying secretive animal behaviors.

## Introduction

Resource allocation is a central theme in behavioral ecology, and life history theory is grounded in such trade-offs (Williams 1966; Stearns 1992). Parental food allocation that maximizes an adult's lifetime reproductive success may fall short of what is required for the offspring to maximize its own lifetime reproductive success (Trivers 1974). For marine organisms, the uncertain nature of pelagic resources in space and time results in unpredictable patterns of foraging success (Mori and Boyd 2004; Weimerskirch 2007; Ronconi and Burger 2008; Carter et al. 2016) and therefore the energy available for offspring.

Procellariiform seabirds travel hundreds or thousands of kilometers from their nesting islands to forage before returning to the nest to feed their single chick (Catry et al. 2009; Froy et al. 2015; Hedd et al. 2018; Mauck et al. 2023). These pelagic seabirds are central place foragers that must balance their own energy needs with those of their chick while navigating environmental uncertainty throughout the extended period of chick development (Chaurand and Weimerskirch 1994; Froy et al. 2015; Tyson et al. 2017). Two key processes interact to determine how much food reaches a hungry chick. First, foraging success dictates the amount of food a parent can acquire. Second, parental allocation decisions determine how much of that food is ultimately provided to the chick, rather than retained for a parent's own needs.

Parental input in mammals and birds is often measured through variation in offspring weight. In brown bears, the duration of maternal care is positively correlated with yearling mass (Van de Walle et al. 2021). Dusek et al. (2017) experimentally manipulated maternal body condition in laboratory mice and measured change in offspring weight every 4 d as an index of parental investment. Similarly, Martin et al. (2023) used offspring birth weight to evaluate maternal energy allocation in gazelles. In birds, fledgling mass and chick weight gain during the nestling period are commonly used as indicators of parental effort (eg, Buchanan and Catchpole 2000; Grzedzicka 2018).

Seabird researchers, in particular, often measure chicks at regular intervals and use the change in mass as an index of parental input (Granadeiro et al. 1998; Schultz and Klomp 2000; Weimerskirch et al. 2000; Thys et al. 2021; Tyson et al. 2022). Once hatched, and after a short period of brooding, the single chick characteristic of procellariiform seabirds is left alone at the nest for weeks or months while parents feed it intermittently every few days (Warham 1990). Thus, researchers can weigh chicks once per day without disturbing the parents and use the daily weight change to assess whether none, 1, or 2 parents have fed the chick in the previous 24 h (Ricklefs et al. 1985; Phillips and Hamer 2000; Gladbach et al. 2009; Haussmann et al. 2024). With the advent of radio frequency identification (RFID) technology (Bonter and Bridge 2011) researchers are able to identify individual parents on the nights a chick was fed, providing a clearer picture of individual adult input from the 24-h weight change when fed. On nights when both parents attend the nest, RFID identities are known but how much of the 24-h mass change can be attributed to which adult has remained unknown (Tyson et al. 2017, 2022). Additionally, 24-h weight change in chicks does not tell us how heavy the parent was when it returned to the burrow after foraging. Thus, measuring chick weights alone cannot distinguish between variation in foraging success and variation in parental allocation decisions.

To address this issue, we developed the Burrow Scale Monitor (BSM) to provide new data on adult mass as a parent arrives and departs while provisioning chicks. The BSM is based on technologies first described by penguin researchers (Kerry et al. 1993; Afanasyev et al. 2015) that we have adapted for burrow-nesting Leach's storm-petrels (Procellariiformes: Hydrobatidae: *Hydrobates leucorhous*). The device allows us to passively estimate the mass of an adult storm-petrel as it enters and exits the nesting burrow, and, therefore, the amount of food delivered to the chick by an individual adult. This method enables a better understanding of how parents allocate the food acquired during their foraging trips between their offspring and themselves.

Similar to other seabirds (Bertram et al. 1996; Gwiazda et al. 2017), food delivery to storm-petrel chicks, as calculated from 24-h mass-changes, tends to be smaller during the initial stages of chick development (Ricklefs et al. 1985; Pollet et al. 2019) and is probably limited by a small chick's ability to receive and assimilate food. This pattern of allocation may result from parental behavior while foraging at sea, in which the stored food in the proventriculus at the end of the foraging trip is matched to the age of the chick (Hypothesis 1). Alternatively, parents may keep their foraging strategies the same, but allocate relatively more of their resources to the chick upon arrival to the nest (Hypothesis 2).

Before addressing these competing hypotheses, we first characterized the variation in adult mass upon return from foraging and its effect on food allocation to the chick without regard to chick age. Given the uncertain nature of pelagic resources, we expected variation in the mass of birds returning to the nest, regardless of chick age. Because the amount of food delivered to the chick is likely constrained by how much food the parent can garner while foraging, we further predicted that *mass delivered* should increase with *entry mass*. While *mass delivered* directly benefits the chick, the *proportion of entry mass delivered* reflects how much of the parent's foraging success is allocated to the chick. Regardless of chick age, it is possible that parents simply give a set percentage of their foraged food to the chick. If so, then the *proportion of entry mass delivered* should not be correlated with *entry mass*. Alternatively, if adults always reserve some minimum amount for themselves, but above that, can give varying amounts to chicks, we might expect *mass delivered* to remain relatively constant across most of the observed range of *entry mass*. Finally, if retaining too much food for the return flight is disadvantageous, we expect that the more food a parent acquires, the more they will allocate to the chick, causing *proportion of entry mass delivered* to increase with *entry mass.*

**Fig. 1.**
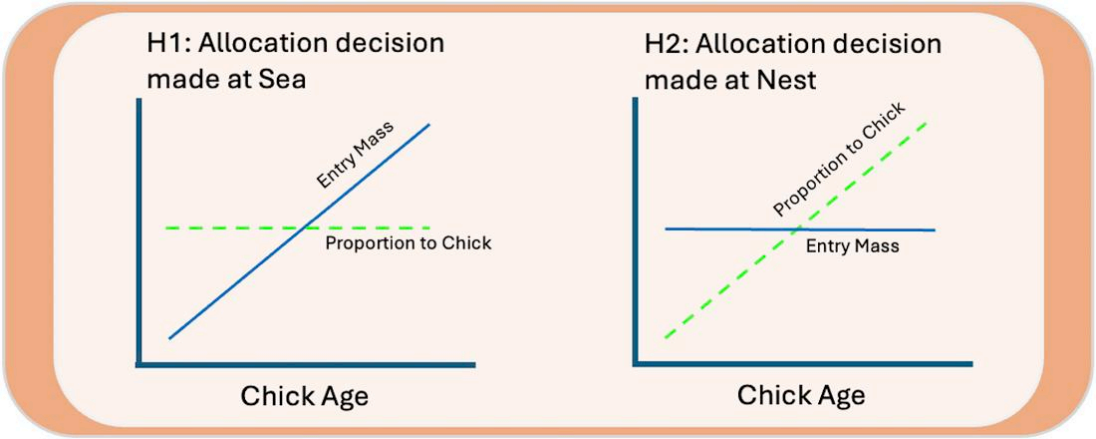
Predictions from competing hypotheses for parental food delivery relative to chick age. If parents adjust entry mass (solid line) to chick age (Hypothesis 1), then entry mass will increase with chick age and the proportion of entry mass delivered (dashed line) will not. The opposite is true if parents forage without regard to chick age and adjust the proportion of entry mass delivered to chick age upon return from foraging (Hypothesis 2).

Within this context, we tested our main hypotheses, ie, whether parents adjust their behavior for chick age at sea (Hypothesis 1) or at the nest (Hypothesis 2). If parents adjust their foraging decisions at sea to chick age, then *entry mass* should increase with chick age through the first 30 d of chick growth, whereas the *proportion of entry mass delivered* should remain stable over that period (Hypothesis 1; Fig. 1). Alternatively, if parents forage without regard to chick age and adjust food delivery upon arrival at the nest, then we predict that *entry mass* will remain unchanged with chick age, but the *proportion of entry mass delivered* will increase with chick age as chicks develop a greater capacity to process food (Hypothesis 2).

## Materials and methods

### Study species and population

**Fig. 2.**
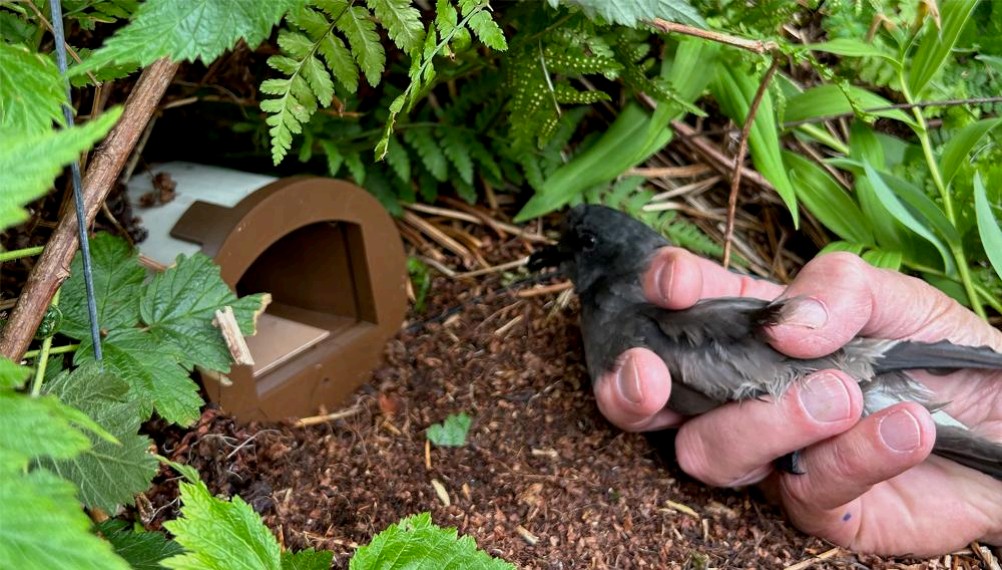
Storm-petrel being released into a BSM during validation trials (Supplementary material S5).

Leach's storm-petrels are the most widespread procellariiform species breeding in the Northern Hemisphere (Pollet et al. 2019). Storm-petrels breed in the North Atlantic after spending the winter in the equatorial Atlantic (Pollet et al. 2014). These seabirds produce a single egg per year for up to 38 yr (Mauck et al. 2012). We conducted this study at a breeding colony of about 25,000 pairs (d’Entremont et al. 2020) of Leach's storm-petrels at the Bowdoin Scientific Station on Kent Island, New Brunswick, Canada (44°35′ N, 66°45′ W) near the mouth of the Bay of Fundy. Although a subset of the breeding population on Kent Island has been studied continuously since 1953 (Mauck et al. 2018), our study sample was drawn from a previously unstudied area on Kent Island encompassing 90 previously unexamined burrows Fig. 2).

Leach's storm-petrel parental care lasts nearly 3 mo with a 42.6-d (±4.5 SD) incubation period, followed by 67.1-d (±4.6 SD) chick-provisioning period (Pollet et al. 2019). On Kent Island, the earliest recorded hatch date was on 4 July and 95% of eggs hatch by 28 August (median = 20 July) based on historical records (R.A. Mauck, unpublished data). Foraging adult males travel an average of 1,627 km (±737.6) per trip during the chick-rearing period, while females travel somewhat less (1,273 ± 688 SD; Mauck et al. 2023). Parents consume planktonic invertebrates and small fish which are processed and stored as energy-rich oils in the proventriculus for delivery to the chick (Place et al. 1989). Both parents independently return to the nest strictly at night, such that chicks up to 10 d old are fed approximately every 1.7 d, with the interval increasing to 2.2 d for older chicks (Pollet et al. 2019). Parents feeding a chick spend anywhere from minutes to hours with the chick before returning to sea (R.A. Mauck, unpublished data). Chicks hatch at about 10 g and quickly grow to exceed average adult mass (∼45 g), reaching a mean maximum weight of 71.2 g (±7.3 SD) before losing sufficient weight to fledge (Mauck and Ricklefs 2005).

### Burrow Scale Monitor

The BSM is installed in the burrow entrance (Fig. 2) and allows us to passively estimate the mass of an adult storm-petrel as it travels through the device. The design for the BSM was inspired by the penguin weighbridge (Kerry et al. 1993) in that it employs a platform suspended on a load cell over which a bird walks while going to and from the nest. The design and construction of the BSM are described in Supplementary material S1 and S2, as are the details of device deployment in the field (Supplementary material S3). We wrote custom software (Supplementary material S4) to convert the raw load cell data produced by the BSM to mass (g). We validated the efficacy of the BSM by testing it against known-weight birds under different conditions (Supplementary material S5).

We then performed power analyses of the BSM-based measurements given the degree of error found during that testing. These analyses indicated that BSM-based measures are robust in detecting underlying differences between 2 groups of adults feeding chicks under normal circumstances (Supplementary material S6). Although our design is tuned to storm-petrels, it could be adapted to a wide range of burrow-dwelling wild species or any species with constrained movement patterns that can require them to pass over a platform.

### Field methods

We first installed 15 BSMs into the burrow entrances of incubating birds between 24 June and 5 July 2025 (14 July ± 4.4 d, mean + SD), early enough to have the units operating when the chick was hatched. A 16th BSM was installed on 22 July in a burrow with an unknown hatch date. This burrow was not included in any analyses in which chick age was an independent variable. Since the study area had not been previously monitored, we had no past information on any individual storm-petrel parent. Burrows were chosen such that the anticipated hatch date would be early enough for us to track a significant number of chick-feeding events with the BSM before the field season ended. Hatch date was estimated by gauging the date the egg was laid after Haussmann and Mauck (2007). A second criterion was that the burrow entrance must be within 15° of level for optimal BSM operation. The focal burrows were chosen at random from the subset of qualifying burrows and we had no reason to believe these burrows differ in any significant way to those of the general population.

We attached RFID tags (Bonter and Bridge 2011) to leg bands of both adults in the burrow and placed RFID readers at each burrow entrance. We fitted each individual with a USFWS band on the other leg and measured flattened wing chord (WL, mm). Hatch dates of the eggs in the 15 burrows with known hatch dates ranged from 9 July to 3 August. BSM data collection ended on 9 August, which allowed us to measure adult entry and exit masses for chicks across the initial 20.8 ± 7.3 d of chick rearing for those 15 burrows.

Each day, the system was reactivated and calibrated by briefly placing 3 known weights on the platform at the burrow entrance (Supplementary material S3). The system then ran through the next ∼24 h, recording overnight visits by adults, until the next day when the system was deactivated, and the data were downloaded.

### Statistical analyses

We focus on 4 variables: 2 direct measures (*entry mass* and *exit mass*) and 2 derived measures (*mass delivered* and *proportion of entry mass delivered*). To evaluate our focal variables, we prepared the field data as described in Supplemental material S5. We used the resulting adult *entry mass* (*M_Entry_*) and *exit mass* (*M_Exit_*) to calculate *mass delivered* (*M_Del_*) and *proportion of mass delivered* (*P_Del_*) in this way:


$$P_{Del}=\left(\frac{M_{Del}}{M_{Entry}}\right)\;\mathrm{where}\;M_{Del}\;=\;(M_{Entry}\;-M_{Exit})$$

**Fig. 3.**
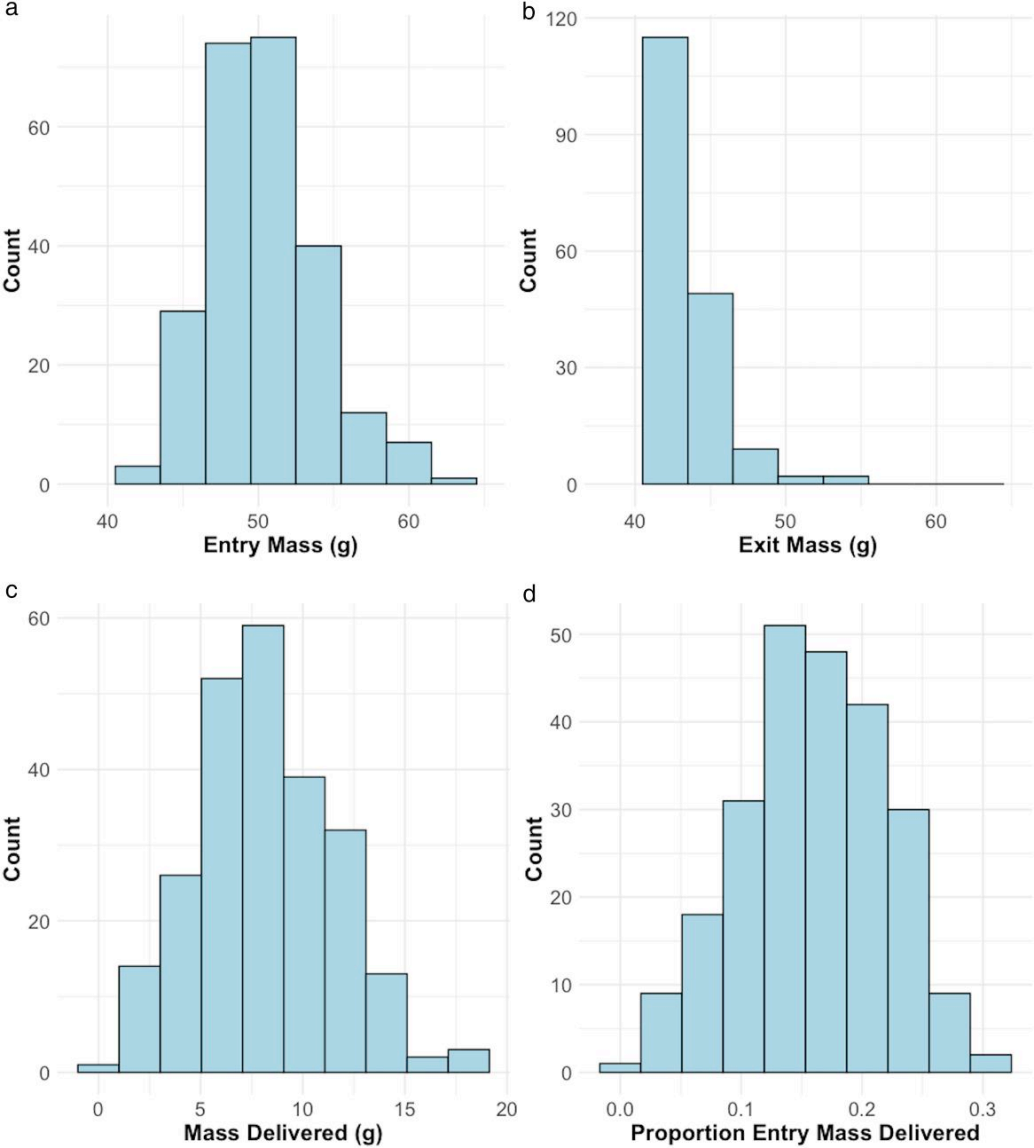
Distributions of entry mass (a; *M_Entry_*), exit mass (b; *M_Exit_*), mass delivered (c; *M_Del_*), and proportion of entry mass delivered (d; *P_Del_*) of adult storm-petrels during 241 identified feedings, as measured with a BSM on Kent Island between 9 July—9 August 2024.

All analyses were conducted in R version 4.1.1 (R Core Team 2021). We used linear mixed effects modeling (“lmer”) with the package “lme4” (Bates et al. 2015) to estimate the relationships between variables in the field data (chick age, $M_{Entry}$, $M_{Del}$, $P_{Del}$). All models involved normally distributed response variables ($M_{Entry}$, $M_{Del}$, $P_{Del}$; Fig. 3). We used linear mixed models (LMM) fit by restricted maximum likelihood (REML) with the default “identity” link. Within the LMMs, we used Satterthwaite's method in “lmerModLmerTest” to calculate the *t*-value of the independent variables (Kuznetsova et al. 2017). We inspected residuals of all models to confirm the assumptions of the model and only comment if they were not met.

Our analyses involved a multi-step process. First, to understand the context in which parents make allocation decisions, we examined food delivery without respect to chick age. Specifically, we constructed a series of “context” models using all 16 burrows (Table 1) to test hypotheses regarding the variation in food delivery given the uncertain nature of pelagic food sources. We then constructed a model to confirm that food delivery to the chick increased with chick age (Table 2, model 1). Next, we constructed models to test our main hypotheses regarding parental allocation decisions (Table 2, models 2 and 3). Once we determined which of the competing hypotheses best fit the data, we tested whether the background context of foraging success was sufficient to explain food delivery to a chick (Table 2, model 4) and employed a likelihood ratio test (LRT) to determine whether the addition of chick age significantly improved the fit. For these models, we used the full dataset of BSM-based food deliveries in the 15 burrows for which chick age was known (*N* = 240). Because the RFID system had intermittent failures, we could not establish individual adults (via RFID tag) for all the weights recorded by the BSM. Thus, for the full dataset, we accounted for the non-independence of deliveries to specific chicks in each model by including “Burrow” as a random effect. Once the best model was identified to address our main question, we then used the subset of food deliveries (*N* = 169) with associated RFID values to determine whether that best model structure fitted with a random effect of “RFID” nested within “Burrow” yielded the same qualitative result (Table 2, model 5) when individual identity was known. We also included wing length (WL; mm) in this last model to determine whether the adult size improved our understanding of food delivery. Finally, we used the smaller subset of data (*N* = 115) for which we could reliably measure time between feedings by individual adults to investigate whether trip length affected our hypotheses. Thus, we added *interval* to the 3 models testing our main hypotheses (Table 2, models 2 to 4) to build the same 3 models with the added variable *interval* (Table 2, models 6 to 8) to understand how trip length affected food delivery and chick age.

**Table 1.** Context models.

Model	Dep var	Fixed eff	Random eff	*N*	*Est*	*SE*	*df*	*t*	*P*	Sig.
1.1	*M_Del_*	*M_Entry_*	Burrow	241	0.69	0.04	232.5	16.5	<2E−16	Yes
1.2	*P_Del_*	*M_Entry_*	Burrow	241	0.01	0.008	231.2	12.73	2.00E−16	Yes
1.3	*P_Del_*	*M_Del_*	Burrow	241	0.02	0.002	231.2	99.41	<2E−16	Yes

Each line represents an LMM to test hypotheses about the background variation without respect to chick age.

*M_Del_* represents mass of food delivered to chick. *M_Entry_* represents mass of the adult when it entered the burrow. *P_Del_* represents the proportion of the adult's entry mass delivered to the chick. *Dep Var* represents the dependent variable in the LMM. *Fixed Eff* represents the independent variable in the LMM. *N* is the sample size. Details of the fixed effect are estimated by *Est* its *SE*, along with *df*, *t*, and *P*-value for the fixed effect in the LMM. The last column denotes where or not the effect was significant given our Bonferroni correction (0.004).

**Table 2.** Main hypothesis models.

Model	Dep var	Fixed	Random	*N*	*Est*	*SE*	*df*	*t*	*P*	Sig.
2.1	*M_Del_*	Chk age	Burr	240	0.17	0.03	232.0	5.50	8.18E−08	Yes
2.2	*M_Entry_* (H1)	Chk age	Burr	240	0.08	0.03	234.3	2.48	0.014	NS
2.3	*P_Del_* (H2)	Chk age	Burr	240	0.03	0.0005	233.0	5.70	3.60E−08	Yes
2.4	*P_Del_* (H2)		Burr	240						
		*M_Entry_*			0.01	0.0008	228.5	12.40	2.20E−16	Yes
		Chk age			0.002	0.0004	237.0	5.11	6.53E−07	Yes
2.5	*P_Del_* (H2)		Burr:RFID	169						
		*M_Entry_*			0.01	0.001	155.8	10.79	2.20E−16	Yes
		Chk age			0.003	0.005	164.6	6.29	2.82E−09	Yes
		WL			−0.001	0.002	20.2	−0.43	0.59	NS
2.6	*M_Entry_* (H1)		Burr:RFID	114						
		Chk age			0.08	0.05	110.4	1.82	0.07	NS
		Interval			0.50	0.20	110.7	2.45	0.02	NS
2.7	*P_Del_* (H2)		Burr:RFID	114						
		Chk age			0.003	0.001	110.5	3.97	1.29E−04	Yes
		Interval			0.013	0.004	110.3	3.54	5.85E−04	Yes
2.8	*P_Del_* (H2)		Burr:RFID	113						
		*M_Entry_*			0.013	0.001	108.9	11.04	2.00E−16	Yes
		Chk age			0.002	0.001	108.6	3.31	1.29E−03	Yes
		Interval			0.006	0.003	108.6	2.38	0.019	NS

Horizontal lines separate the details of a single LMM that addresses either Hypothesis 1 (H1; decision made at sea) or Hypothesis 2 (H2; decision made a nest). Models with more than 1 fixed effect, have a line for each of those fixed effects. *M_Del_* represents mass of food delivered to chick. *M_Entry_* represents mass of adult when it entered the burrow. *P_Del_* represents the proportion of the adult's entry mass delivered to the chick. *Chk Age* represents Chick Age. *Interval* represents the days at sea before the current feeding. Adult wing length is denoted by *WL*. *Dep Var* represents the dependent variable in the LMM. *Fixed Eff* represents the independent variable in the LMM. *N* is the sample size. Details of the fixed effect are estimated by *Est* its *SE*, along with *df*, *t*, and *P*-value for the fixed effect in the LMM. The last column denotes whether (Yes) or not (NS) the effect was significant at the level of our Bonferroni correction (0.004).

While chick growth as indexed by wing length in storm-petrels follows a sigmoidal curve (Mauck and Ricklefs 2005), Kent Island historical measures of 24-h weight change by <30 d old chick does not (*R*^2^ = 0.17, R.A. Mauck, unpublished data). We, therefore, did not a priori test for polynomial effects on chick age.

Because we performed 3 LMMs to address the background context (Table 1), and 8 LMMs and 1 LRT to address the main hypothesis (Table 2) of this paper, we evaluated the statistical significance of our results by applying a Bonferroni correction (alpha = 0.05/12 = 0.004).

## Results

We installed BSMs in 16 burrows that contained chicks, covering a total of 328 burrow-nights. We recorded 264 identifiable feeding events between 8 July and 9 August 2024. Those 264 feeding events occurred on 204 burrow-nights (1.3 feedings/burrow-night when fed). After excluding feeding events for which either *M_Entry_* or *M_Exit_* fell outside the Kent Island historical 0.1 percentile thresholds (38.9 g, 61.0 g; Supplementary material S5) and, therefore, were more likely to be measurement error than biological extremes, we used the remaining 241 measurements of *M_Entry_* (50.4 ± 3.6 mean ± SD g; Fig. 3a) and *M_Exit_* (42.3 ± 2.6 mean ± SD g; Fig. 3b) to calculate *M_Del_* for each feeding event (8.1 ± 3.5 mean ± SD g, Fig. 3c) as well as *P_Del_* (0.16 ± 0.06 mean ± SD; Fig. 3d). Both $M_{Del}$ and *P_Del_* showed greater variation (Levene *F* > 3,300, *df* = 239) than *M_Entry_* (CV = 7.1).

**Fig. 4.**
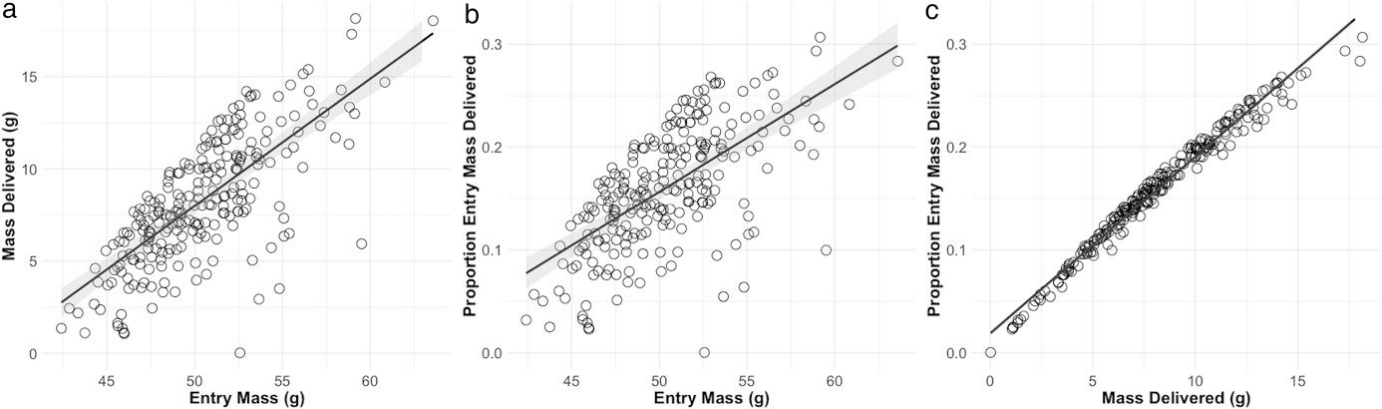
Background context relative to mass delivered (*M_Del_*), entry mass (*M_Entry_*), and proportion of entry mass delivered (*P_Del_*). The effect of *M_Entry_* on *M_Del_* (a) and on *P_Del_* (b). The effect of *M_Del_ on P_Del_* (c). Based on GLMM models with Burrow (*N* = 16) as a random effect for 241 identified feedings by adult storm-petrels as measured with a BSM on Kent Island between 8 July—9 August 2024. Shaded areas represent the 95% confidence interval (N.B., the 95% CI for panel c is extremely narrow).

Without regard to the age of the chick, LMMs showed strong relationships between *M_Entry,_ P_Del_*, and $M_{Del}$. Food delivered to the chick ($M_{Del}$) increased with *M_Entry_* (Table 1, model 1; Fig. 4a). *P_Del_* also increased with *M_Entry_* (Table 1, model 2; Fig. 4b). *P_Del_* most strongly correlated with $M_{Del}$ (Table 1, model 3; Fig. 4c). These relationships demonstrate that food delivery to the chick, whether $M_{Del}$ or $P_{Del}$ is constrained by how much the adult weighs on return from foraging.

**Fig. 5.**
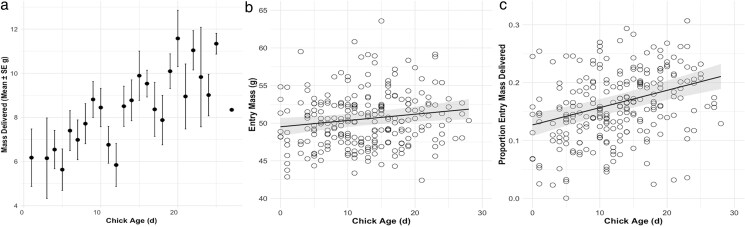
Mass delivered (mean ± SE) increases with chick age (a). Results of LMMs of the effect of (b) only chick age on *M_Entry_* and (c) only chick age on *P_Del_*. Solid line shows the estimate of the effect of chick age on the dependent variable. The shaded area represents the 95% CI.

To test our main hypothesis, we first confirmed that $M_{Del}$ increased with chick age (Table 2, model 2.1; Fig. 5a). Using the full dataset of measurements for which we knew chick age, we then tested the predictions from our competing hypotheses. As predicted by Hypothesis 2 (Fig. 1), chick age did not significantly influence *M_Entry_* (Table 2, model 2.2; Fig. 5b) but did significantly influence *P_Del_* (Table 2, model 2.3; Fig. 5c).

Given the strong influence of parental *entry mass* on food delivered to the chick (Fig. 4a and b), it is possible that entry mass alone was sufficient to explain variation in *proportion of entry mass delivered*. Therefore, to better understand the effect of chick age on *P_Del_* we tested whether a model that includes only parental *M_Entry_* was sufficient to explain the *proportion of entry mass delivered* to the chick or whether the model could be significantly improved by including chick age (Table 2, model 2.4). In this improved model, both chick age and parental *entry mass* were significant predictors of the *proportion of entry mass* delivered to the chick (Table 2, model 4). Collinearity between parental *entry mass* and *chick age* was negligible (VIF = 1.02), indicating that both variables contribute independently to the model. When tested against a model with only *entry mass* (Table 1, model 1.1 using only burrows known-age chicks), the model that included *entry mass* and chick age showed a significantly better fit (LRT, *χ*^2^ = 24.1, *df* = 1, *P* = 9.1e^−07^) suggesting that within the constraints of entry mass, the *proportion of entry mass* delivered to the chick increases with chick age as predicted by Hypothesis 2.

We then re-tested our main hypotheses using subsets of the full dataset in which we knew the identity of individual adults and therefore included RFID nested within Burrow as our random effect. First, we investigated the influence of adult size by adding wing length to model 2.4. This new model (Table 2, model 2.5) showed no effect of WL with an even stronger effect of chick age. We then repeated our initial tests related to Hypothesis 1 (Table 2, model 2.2) and Hypothesis 2 (Table 2, model 2.3) but added the *interval* between feeding events to those models (Table 2, model 2.6, 2.7, 2.8). A simple Kendall rank correlation suggested that *interval* increased with chick age (Kendall *τ* = 0.23, *t* = 3.15, *P* = 0.002, *n* = 114) and *interval* was positively correlated with *proportion of entry mass* delivered to the chick in the simpler model (Table 2, model 2.7), but not in the final model (Table 2, model 2.8) that included *entry mass*. There was no evidence of collinearity (VIF < 1.3) among the multiple fixed effects in any of these models. As before, even when *interval* was included, chick age did not significantly explain variation in *entry mass* (Table 2, model 2.6). However, chick age did significantly explain variation in the *proportion of entry mass* delivered to the chick (Table 2, models 2.7 and 2.8), again supporting Hypothesis 2 over Hypothesis 1.

## Discussion

Storm-petrel parents return to the nest without any apparent adjustment of arrival weight based on chick age, yet they deliver larger loads as their chick gets older. Adult arrival weight varies widely, and the heavier the bird upon arrival, the more it can download to the chick; this relationship holds independently of the chick age and accounts for most of the variation in food delivered during the first 30 d of chick rearing. However, adults also deliver more food to older chicks over this period. This increase stems to a much greater extent from parents delivering a greater proportion of their body mass as food (ie, a shift in parental allocation; Hypothesis 2), rather than from adults increasing their body mass before arrival (ie, a shift in foraging strategy; Hypothesis 1).

While we found no support for Hypothesis 1, food delivered to the chick was positively correlated with the interval between an individual adult's feeding visits. However, models that included this interval with chick age still supported Hypothesis 2 and not Hypothesis 1 and when both chick age and entry mass were included, interval was not significant. Many species of procellariform birds have been shown to utilize a dual-foraging strategy thought to balance offspring benefit with self-benefit (Weimerskirch and Jouventin 1993; Phillips et al. 2023). Shorter intervals between trips provide the chick with smaller, more frequent feedings at a cost to the parent (Cuthill and Kacelnik 1990; Weimerskirch 1998; Weimerskirch and Cherel 1998), whereas longer trips provide less frequent, larger loads but result in improved adult condition (Weimerskirch et al. 1994; Granadeiro et al. 1998). Many seabird studies have shown foraging trip duration is sensitive to adult condition and prey availability (Burke and Montevecchi 2009; Ballard et al. 2010; Daenhardt et al. 2011). At Kent Island, it has been shown that parents respond to chick hunger state through trip frequency and chick 24-h weight gain increases with interval between feedings (Haussmann et al. 2024). Trip duration might be considered adjustment at sea, but if so, Hypothesis 1 should have been supported. Perhaps, the allocation of foraging time is a decision on a different temporal scale than the decision supported by Hypothesis 2 which is made after the foraging trip has been completed.

The fact that adult wing length was not a significant factor explaining food delivery is not surprising. On Kent Island, the mass of incubating adults is not correlated with adult wing length when the adult is measured within a few hours of returning to the nest (R.A. Mauck, unpublished data). The mean mass of those birds (51.2 ± 3.9 g SD, *N* = 856, from 2003 to 2017, R.A. Mauck, unpublished data) is similar to the mean mass of individuals returning to the nest in this study when factoring in the mean error of the BSM (50.4 g + 0.7 g = 51.1 g). The maximum adult mass among 3,887 incubating individuals that have been weighed while incubating on Kent Island between 2001 and 2016 (68 g) may be close to the maximum mass at which a storm-petrel can fly. Assuming an average adult storm-petrel mass without food in the proventriculus of 45 g (Pollet et al. 2019), that 68 g individual may have arrived with a 23 g load, somewhat larger than the maximum food delivery to the chick of 18.2 g found in this study.

Given the results of our validation efforts, the agreement between the historical measurements and our BSM measurements was to be expected. Our validation procedure also included a simulation which indicated sufficient statistical power to detect meaningful underlying differences between 2 groups of 15 individuals even with the device-error associated with the BSM. Our tests of the competing hypotheses did not suffer from a lack of power; the relationships were clear. A larger sample size would not have changed the relative support for Hypotheses 1 and 2.

The strong support for Hypothesis 2 over Hypothesis 1 might be expected in a long-lived species such as storm-petrels, where the balance between current and future reproduction tends to favor the parent's long-term survival (Stearns 1992). Given that the pelagic nature of their food sources necessitate long journeys to ephemeral feeding patches, often unpredictable in space and time (Robert et al. 2015; Cerveira et al. 2020; Madden et al. 2023), it may not be advantageous for an adult to adjust foraging strategy based on short-term chick needs at the nest. Indeed, experiments have shown that procellariiform parents tend to prioritize self-maintenance over offspring provisioning when the cost of reproduction is artificially increased (Ricklefs 1987; Mauck and Grubb 1995; Tveraa et al. 1997; Navarro and González-Solís 2007; Ogawa et al. 2015), though recent work in this species suggests that this might not always be the case (Haussmann et al. 2024).

The fact that food delivered per trip increased with chick age does not necessarily mean that the parent “decided” to allocate a smaller proportion of its entry mass to the young chick. It may be that a small chick (<15 g) is simply unwilling or unable to accept a large bolus of regurgitated food, and therefore the parent is not permitted to offload a larger amount. At present, what we do know is that, in general, a parent attending a young chick left a smaller proportion of its arrival mass at the nest than did a parent encountering an older, larger chick.

The fundamental trade-off faced by parents between allocation to self vs. allocation to offspring requires that a balance must be struck between these competing demands (Trivers 1974). Our results suggest that this balance point is not fixed, but shifts over the course of chick development—highlighting how the quantitative nature of the trade-off between parental and offspring needs can change dynamically within the lifespan of not only parents, but also offspring (Taylor and Prum 2024).

Measuring this balance is challenging, as it is difficult to identify a common currency with which to quantify the costs and benefits of this foundational trade-off. Quantifying the benefits of parental behavior to offspring is relatively straightforward in many species. Metrics such as egg size in birds (Velando et al. 2006), offspring structural growth or condition in birds (Schoepf et al. 2022) and mammals (Martin et al. 2023), and survival to independence (Frigerio et al. 2021; Bădescu et al. 2022) all reflect the outcomes of resource allocation to offspring. These metrics are indicators of parental investment but do not assess the cost to the parent, aside from the implied cost of acquiring those resources.

Parental costs have been quantified in a variety of ways. Loss of body mass in breeding adults has served as a proxy for parental costs across taxa, including birds (Guillen-Parra et al. 2024) and mammals (Dušek et al. 2017). Glucocorticoid hormone levels reveal more subtle physiological costs in mammals (Havenstein et al. 2021) and birds (Chastel et al. 2005). Researchers have also measured the energetic cost of parenting using doubly labeled water respirometry, and accelerometry, particularly in birds (Tieleman et al. 2008; Gremillet et al. 2018; López-López et al. 2022), but also in mammals (Kenagy et al. 1990; Jeanniard-du-Dot et al. 2017) and other taxa (Clarke et al. 2021). Newer cellular measures, such as oxidative stress and telomeres are emerging as key integrative mechanisms for understanding the life history strategy (Haussmann et al. 2003; Wilson et al. 2012; Constantini 2014; Colominas-Ciuro et al. 2017; Tricola et al. 2018; Stier et al. 2019; Costantini 2024). These studies often pair measures of parental cost with measures of offspring benefit to capture the nature of the trade-offs, though they typically rely on different currencies to do so.

The BSM enabled us to directly measure 1 parent-offspring trade-off using a common currency: the opportunity cost of stored energy. A parent arrives at the nest with a proventriculus full of food. How much of this stored energy should be retained for self-maintenance, and how much should be transferred to the chick? Food retained by the parent may serve as a reserve for future self-maintenance or fuel the next foraging trip (Beaulieu et al. 2010). In contrast, food delivered to the chick is positively correlated with overall chick growth trajectory (Mauck and Ricklefs 2005). The BSM allowed us to isolate a single parenting decision where both the cost and benefit to the parent's lifetime reproductive success can be measured in the same units, stored energy. Thus, the BSM allowed us to examine a question that to our knowledge was previously unexplored.

Although our study focused on how this trade-off shifted with chick age, BSM-derived data could be used to explore how parental investment varies with adult condition or changing foraging conditions at sea. With accurate measurements of adult departure mass, return mass, and energy expended during foraging, it would be possible to calculate energy gained while foraging as a function of energy expended, ie foraging efficiency (Grémillet 1997; Saraux and Chiaradia 2022). In combination with cellular and physiological measures, this approach could provide new insights into individual variation in reproductive behavior and life history strategies.

## Supplementary Material

araf127_Supplementary_Data

## Data Availability

Analyses reported in this article can be reproduced using the data publicly accessible at this link: https://doi.org/10.5061/dryad.z8w9ghxsv.
