# Supplementary material for: Plan ahead, or wing it? How storm-petrel parents adjust food delivery to young chicks
Source: Behav Ecol. 2025 Nov 7;36(6):araf127. doi: 10.1093/beheco/araf127 (PMC12636516; doi:10.1093/beheco/araf127)
Supplement: araf127_Supplementary_Data [file araf127_supplementary_data.docx]

**SUPPLEMENTAL MATERIALS**

*1 Overview of the Burrow Scale Monitor (BSM)*

We developed, deployed, and validated Burrow Scale Monitors (BSM) to automatically measure the mass of adult seabirds as they enter and exit their nesting burrows. This technology was adapted from previous work on penguin weighbridges [(Kerry et al. 1993)](https://www.zotero.org/google-docs/?slf3yD) to fit the unique demands of burrow-nesting birds. Whereas the dimensions of the penguin weighbridge are not constrained by a particular cross-sectional dimension, the BSM must fit inside the entrance to the burrow tunnel that leads to the nest. As a result, the size of the device is a compromise between the size of the bird to be measured and the size of the opening of the burrow tunnel.

The BSM uses one single point load cell suspended in a 3D-printed inner tunnel (Weight Platform), all of which is mounted within an outer tunnel constructed of PVC pipe. The load cell is controlled with an Arduino. Raw data are stored locally on an SD card and processed through a custom Python application. The cost to make one device ranges from $60 to $260 depending on the quality of the components, which is based on the reliability and precision needed. The current design is specific to storm-petrels but could be adapted to a wide range of burrow-dwelling wild species or any species with constrained movement patterns that can require them to pass over a platform.

*2 Hardware Components*

Load Cell - The main hardware component of the BSM is the load cell, a sensor that measures force or weight by using strain gauges arranged in a Wheatstone Bridge configuration to translate a physical distortion (e.g., a bird standing on a platform) into a change in electrical current that can be calibrated into units of mass. The prototype used an Adafruit 1kg load cell (https://www.adafruit.com/product/4540). The final version uses a Flintec 0.3kg PA1 miniature single-point load cell, which is more expensive but allows for improved precision, stability, ruggedness, and weather-resistance (https://www.flintec.com/weight-sensors/load-cells/single-point/pa1). Data are collected at ~60Hz, a high enough sampling rate that should fulfill the Nyquist criterion for accurate measurements [(Chen and Bassett 2005)](https://www.zotero.org/google-docs/?cNa1EG).

Arduino System - The Arduino system is the electronics platform that provides power and control over all hardware components of the system. The prototype version was built on an Arduino Uno R3 (https://docs.arduino.cc/hardware/uno-rev3/) using a load cell amplifier to interface between the load cell and the Arduino. The current version uses an Arduino Uno R4 Minima (https://docs.arduino.cc/hardware/uno-r4-minima/) which provides increased memory and speed. A 16x2 LCD screen provides user-feedback (https://docs.arduino.cc/learn/electronics/lcd-displays/). Arduino code and wiring logic are available on GitHub (<https://github.com/ninthgiant/Burrow_Scale_Monitor>).

Amplifier - The load cell is connected to the Arduino through an amplifier, which boosts the signal and converts it from analog to digital. We used the HX711 amplifier (<https://docs.arduino.cc/libraries/hx711/>) in the original prototype. The current version uses the AD7193, a high-resolution, low-noise, 24-bit Sigma-Delta analog-to-digital converter by Analog Devices (www.analog.com/ad7193_ds). We use the AD7193 because it provides higher resolution, built-in filters, better noise performance and greater stability compared to the HX711. We contracted with Tacuna Systems (tacunasystems.com) to incorporate the AD7193 and a 16x2 LCD screen into a single shield module to eliminate soldering and minimize wiring in the Arduino system.

Power - The BSM is powered by a 2300 mAh iPhone external battery with a USB-C to USB-A cable connection to the Arduino. As configured, the BSM draws approximately 100 - 130 mA (Arduino: 50 – 60 mA, LCD 50 – 70 mA, Load Cell 2 – 5 mA), which allowed us to use standard USB power banks even though they have an “auto-off” feature that will deactivate the device at very low power (~50 - 100 mA). A configuration that did not include the LCD for user feedback would require a more expensive external battery that can override the “auto-off” feature.

Data Storage - Automated measurement devices can quickly accrue vast amounts of data. The volume of data is determined by the sampling rate and write-content of the device, *not* by the rate of biological events monitored. In other words, data storage requirements are determined by the activity of the device, not the activity of the bird.

Our data are stored on 4 GB or 64 GB SD cards connected directly to the Ardunio and manually transferred to a laptop computer every 24-48 hrs. Over a 24 hr period, one BSM would write approximately 95MB of data. Once a device is deployed, data transfer is the most time-intensive aspect of running the device.

Data Format and Time Keeping - We store raw data on an SD card using a data logger shield with a real-time clock (RTC; <http://hiletgo.com/ProductDetail/2157963.html>). Data are written to a comma-delimited text file, each line of which consists of 2 long integers. The first is the measure of the amplified voltage output from the load cell and the second is a UNIX timestamp which is updated every 5 seconds to minimize accessing the RTC. The format of the file name follows this convention: “DL_MO_DY.txt” Where “MO” is the month, “DY” is the day of the month relative to the day the device was turned on. This filename is generated using the RTC when the device is activated at the start of the day.

Weight Platform and Outer Tunnel - The housing for the device involves an inner tunnel (Weight Platform) that houses the load cell and an outer tunnel that keeps the entire device steady within the burrow itself. This configuration measures the mass of the entire inner tunnel and its contents, thus removing any influence of bird positioning as it moves across the Weight Platform. For improved traction, we attach a 1” x 6” strip of self-adhesive sand paper (400 grit; Dura-Gold) to the floor of the Weight Platform. The Weight Platform and associated components (Figure S.1) are built with a non-commercial 3D printer. Files for 3D-printing (3mf) are available on GitHub (https://github.com/ninthgiant/Burrow_Scale_Monitor). The outer tunnel is from 3” ultra-thin-walled PVC pipe from FlexPVC (CL 100 aka SDR41 3” UltraThin Wall PVC; <https://flexpvc.com/cart/agora.cgi>).

Field Housing - The Arduino and Battery are housed in a weatherproof outdoor connection box ([www.sockitbox.com](http://www.sockitbox.com)) set to the side of each burrow. Operational configuration consists of the Tunnel/Weight Platform apparatus in the burrow tunnel connected by a single wire to the Arduino controller in the waterproof container (Figure S.2).

Additional systems - Multiple devices can be deployed along with the BSM in the same field housing at each burrow (e.g., cameras, microphones, tag readers). In this study we added an RFID system using standard RFID components.

*3 Deployment - Burrow selection* - Before fully deploying the device, we took steps to minimize potential disturbance to parent attendance or behavior. Before storm-petrels arrived at the breeding colony on Kent Island (in April), we placed sham BSMs into burrows where we expected breeding birds. A sham BSM mimicked the outer tunnel of the full device, consisting of a single piece of 7” long, 3” diameter #3 PVC pipe with sandpaper strip on the bottom for traction. We left these sham devices in the burrow entrance until it was time to install a working BSM device. Thus, all birds in our dataset had actively decided to lay an egg in a burrow featuring a device, and had time to habituate to the presence of the device.

From this larger set of burrows with sham devices, we selected a subset of burrows for full deployment. Specifically, we selected burrows that were (1) actively incubated and (2) had relatively flat entrances (<15 degree incline/decline) to minimize artifacts from slope on our final measurements. We measured the slope of each burrow entrance using the built-in leveling capabilities of an iPhone 15.

*4 Deployment - Calibration -* To translate electronic readings from the load cell into a mass value (g), the BSM must be calibrated with three known-weight objects each day upon activation. First, the BSM is powered-up and left undisturbed for five seconds. This provides a valid baseline reading before the calibration. Second, a series of three weights is placed on the platform, in order of lightest to heaviest, each separated by a five second period during which nothing is on the weight platform.

In this study, devices ran undisturbed for 24 hours before data were downloaded for conversion and storage. Each time a device was shut down, the calibration procedure was repeated as a redundancy in case the initial calibration was corrupted. After shutdown, we removed the SD card and downloaded the data to a laptop computer for analysis.

*5 Software -* We provide custom software (MacOS) to summarize, visualize, and convert raw BSM output to mass (g). The software (Figure S.3) provides the opportunity to 1) simply view a data file, 2) manually perform the calibration (Figure S.4) and trace conversion (Figure S.5), 3) run an automated calibration and conversion procedure on the file, and 4) process multiple datafiles in the same folder using the automated procedure. We wrote the software in Python version 3.1.2 [(Python Software Foundation 2023)](https://www.zotero.org/google-docs/?exG5TS) with a Tkinter GUI interface (Figure S.3-4; [Moore 2018)](https://www.zotero.org/google-docs/?2nRPfS). Source code is available on GitHub (https://github.com/ninthgiant/Burrow_Scale_Monitor).

To convert raw load cell data to grams, the user chooses a saved file. The raw data are displayed so that the user can identify the calibration procedure. The software then calculates a regression using the baseline and the three known calibration weights (light, medium, heavy). The resulting regression is used to convert all subsequent bird activity on the platform into grams.

The calibration procedure should result in a nearly perfect fit (*r* = 1.0) of the 3 known-weights vs. voltage (Figure S.4c). Once the calibration is completed and any traces have been converted, the data are saved in a text file whose title is in the format “DL_MO_DY.txt” Where “MO” is the month, “DY” is the day of the month relative to the day the device was turned on. NOTE: in practice, post-hoc every downloaded file has the name appended with “_Burr” to link that day’s text file to a specific burrow.

The most basic procedure for calculating bird weights is done manually (Figure S.5). The manual process begins with the calibration process, then involves identifying a flat baseline near each bird event, then identifying start and stop points of each bird event itself, based on the experience of the user. This method allowed the user to identify the most appropriate section to start and stop the calculation. The choice of the most representative section is important to final conversion.

However, this manual procedure is time consuming and sensitive to user experience. Thus, we also developed a more standardized, automated procedure. The automated method selects all relevant stretches of activity (including calibration and bird events) based on an algorithm that identifies the most stable and level section of the focal trace. For most measurements, the automated method is sufficiently accurate, but for the subset of traces that are less stable, accuracy can be improved by use of the manual system (see next section).

*6 Validation -* To determine the accuracy of bird weight measurements by the BSM, we pulled 53 incubating storm-petrels from their burrows between June 20-27, 2024, and quickly walked them through the BSM. The protocol was to 1) weigh the bird to the nearest 0.1g with an Ohaus scale, 2) activate the BSM, 3) calibrate the device with 3 known weights (15.97, 32.59, 50.22g), 4) place the bird in front of the BSM and allow it to pass through the device.

We tested birds under three different BSM conditions to gauge variation in the accuracy of the weight estimation. First, we ran 20 birds through a BSM under highly controlled conditions, in which the device was anchored to a stable platform not connected to a burrow; the exit end of the BSM was enclosed in a dark bag motivating the storm-petrel to pass through the tunnel from sunlight to darkness. Second, we ran 20 birds through a BSM placed in an otherwise empty burrow, allowing for multiple measurements under natural burrow conditions. In both these runs, the focal storm-petrel was captured in its nest, transported to the BSM, then returned to the nest after the trial which consisted of 2-4 passages through the BSM. Third, we ran 13 birds through a BSM inserted into the entrance of each bird’s own burrow. The storm-petrel was left to re-enter its burrow through the BSM, providing a gauge of weighing accuracy under the most natural conditions. These three conditions yielded 120 passages across the 53 known-weight birds.

Initially, data were converted to mass using the manual procedure. The user performed the conversion unaware of the true weight of the bird. Afterwards, the data were also converted to mass using the automatic procedure.

We first graphed the known vs. calculated mass of each measurement using the automated results (Figure S.6a). We noticed a few extreme outliers. We used historical records of adult incubation weights (N = 3887; unpubl.) to identify the upper 0.5th percentile (61.4g) and lower 0.5th percentile (38.7g). We decided to exclude values outside those bounds under the assumption that such outliers were more likely to be measurement error than biologically accurate. Most of the remaining deviation occurred in the lower calculated values. Because manual calculations tend to be more accurate (diff = 0.26 ± 1.5STD g; paired t = 1.74, df = 115, P = 0.08) than automated calculations, we used the manual calculation method results to recalculate any values that fell below the historical 10th percentile of adult incubation weights (43.3g) using the manual method. This improved the fit (Figure S.6.b). Thus, we established our procedure for processing data using the following protocol:

Step 1) calculate all weights using the automatic procedure

Step 2) remove the upper and lower extreme values relative to the historical distribution of adult weights

Step 2) recalculate manually values that fell within the lower 10th percentile of the historical distribution.

Using this protocol, the median device error (Known - Measured) was 0.2g, slightly less than mean error (0.6g ± 1.8 STD). The error distribution (Figure S.7) was slightly skewed (skew = 1.2) toward lower calculated values and showed some kurtosis (kurtosis = 1.8).

*7 Power Analysis -* We used the error distribution from validation to perform a power analysis to determine whether device error interferes with the ability to detect a biological difference between two groups of birds delivering food to the offspring. The simulation allowed us to hypothesize a variety of levels of differences between the groups across a variety of sample sizes in the study.

We wrote a python program that allowed us to test our ability to distinguish between two populations (e.g., males and females) under conditions we could control:

1. Measurement error of the BSM (0.6 ± 1.8 STD g)
2. Group size = number of individuals in each group
3. Feeding Events/Foraging Trips per individual
4. Hypothesized difference between groups (%)
5. Mean and STD, Min and Max of Background Meal Size - from historical data
6. Mean and STD, Min and Max of adult body size- from historical data
7. Number of iterations (> 1000)

These parameters were applied to the logic presented in Figure S.8. Note that the error associated with BSM measurement is applied twice per feeding event, once upon entry and once upon exit. The simulation reports many statistics relative to the output, the most important being what proportion of the iterations reported a significant difference between the two groups. The code is available on GitHub (https://github.com/ninthgiant/Burrow_Scale_Monitor).

The analysis that imposed the observed error (STD = 1.8g) between actual and calculated mass as a bird enters and exits the burrow showed that a difference between 2 groups of individuals should be detectable under normal circumstances (Figure S.9). For example, if we hypothesize that one group (N=15) delivers 20% more food per feeding than the other group (N=15) and we measure 8 feeding events per bird over a single season, a significant difference between the groups was detected 962 out of 1000 times (Probability of Detection = 0.96)

**LITERATURE CITED IN SUPPLEMENTAL MATERIALS**

[Chen K, Bassett D. 2005. The Technology of Accelerometry-Based Activity Monitors: Current and Future. Med Sci Sports Exerc. 37(11).](https://www.zotero.org/google-docs/?DjNhXv)

[Kerry K, Clarke J, Else G. 1993. The use of an automated weighing and recording system for the study of the biology of Adelie penguins (Pygoscelis adeliae).](https://www.zotero.org/google-docs/?DjNhXv)

[Moore AD. 2018. Python GUI Programming with Tkinter. 1st ed. Birmingham: Pakt Publishing Ltd.](https://www.zotero.org/google-docs/?DjNhXv)

[Phillips RA, Hamer KC. 2000. Periodic weighing and the assessment of meal mass and feeding frequency in seabirds. J Avian Biol. 31(1):75–80.](https://www.zotero.org/google-docs/?DjNhXv)

[Python Software Foundation. 2023. Python (Version 3.12). https://www.python.org/.](https://www.zotero.org/google-docs/?DjNhXv)

[Ricklefs RE, Day CH, Huntington CE, Williams JB. 1985. Variability in Feeding Rate and Meal Size of Leach’s Storm-Petrel at Kent Island, New Brunswick. J Anim Ecol. 54(3):883–898.](https://www.zotero.org/google-docs/?DjNhXv)

*Figures*

S.1 - Exploded View of Burrow Scale Monitor


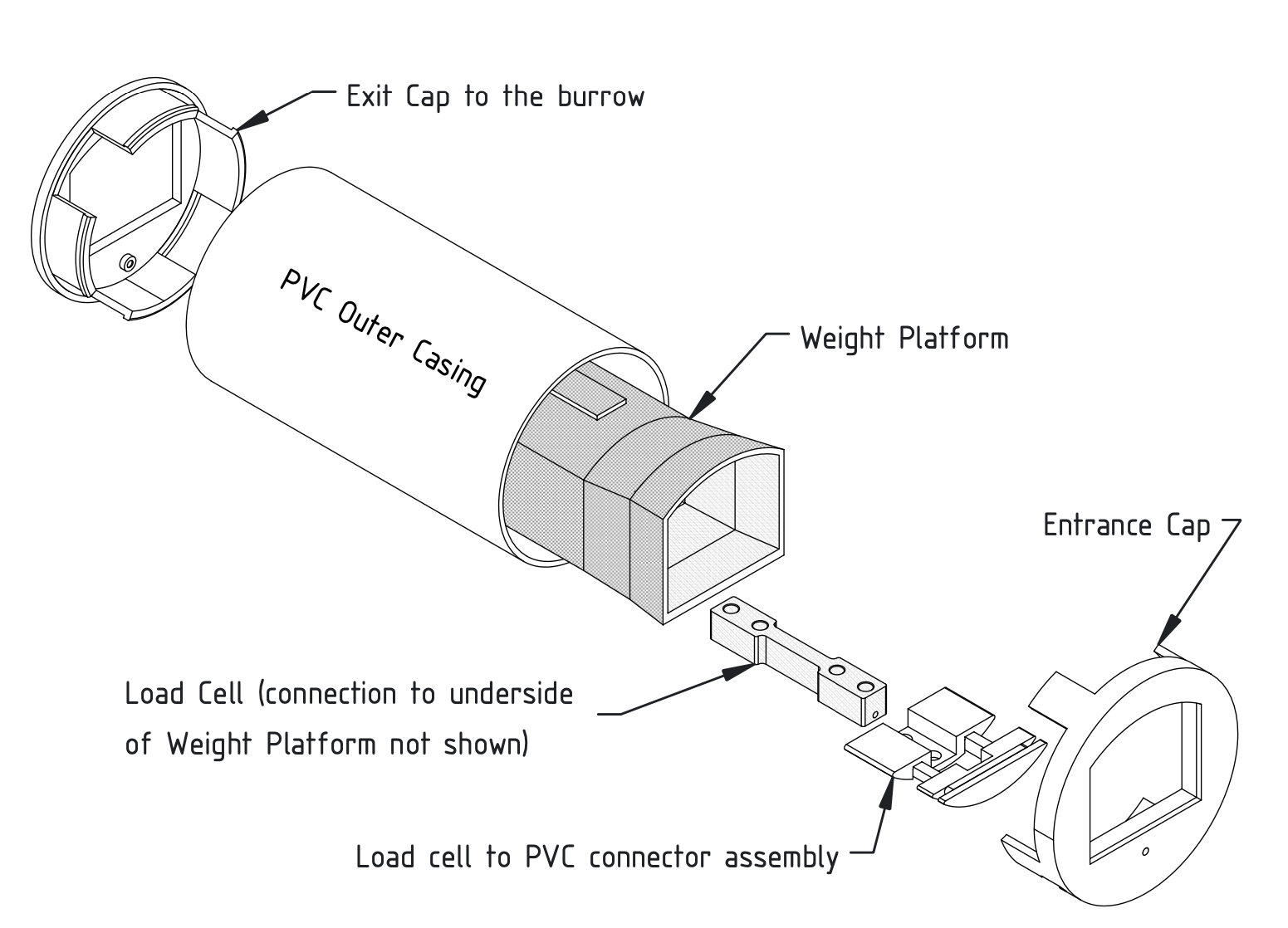


Figure S.1. Exploded view of the tunnel within a tunnel of the BSM. The Weight Platform is suspended within the PVC Outer Casing via the Load cell. One end of the Load Cell is attached to the bottom of the Weight Platform with #4 machine screws. The other end of the Load cell is connected to PVC outer casing through the Connector assembly with #4 machine screws. The connector assembly has 2 parts (Plug and WireClamp). Both ends of the Outer Casing are capped to prevent dirt and debris from entering the tunnel assembly.

S.2 Field Deployment


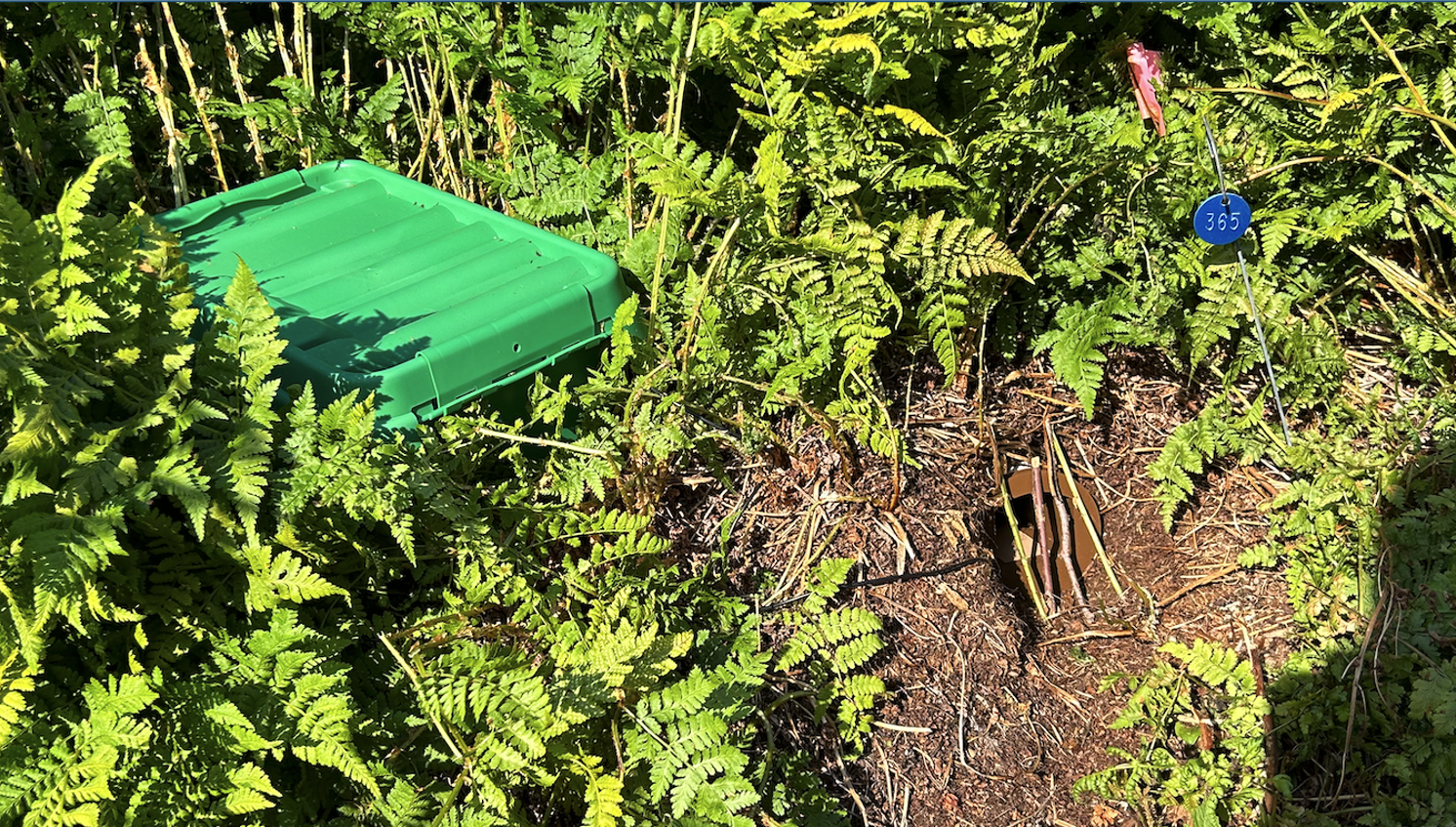


Figure S.2. Field installation. Inside the green waterproof box are the Arduino and the battery. The black wire runs from the Arduino to the BSM tunnel within a tunnel that is inserted in the burrow entrance. The sticks covering the burrow entrance indicate to the user whether the burrow entrance has been disturbed the previous night.

S.3 - GUI Interface


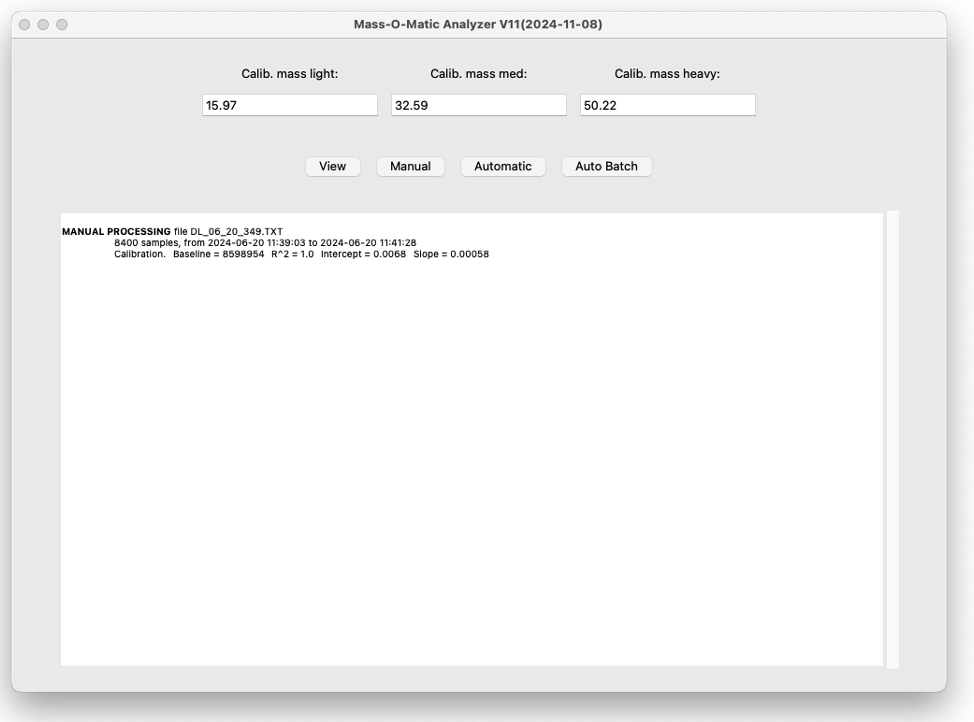


Figure S.3. Initial screen of the python application. The values for the calibration are entered at the top of the screen. Buttons allow the user to choose a function. Information resulting from those choices appear in the text box at the bottom half of the screen.

S.4 - Calibration
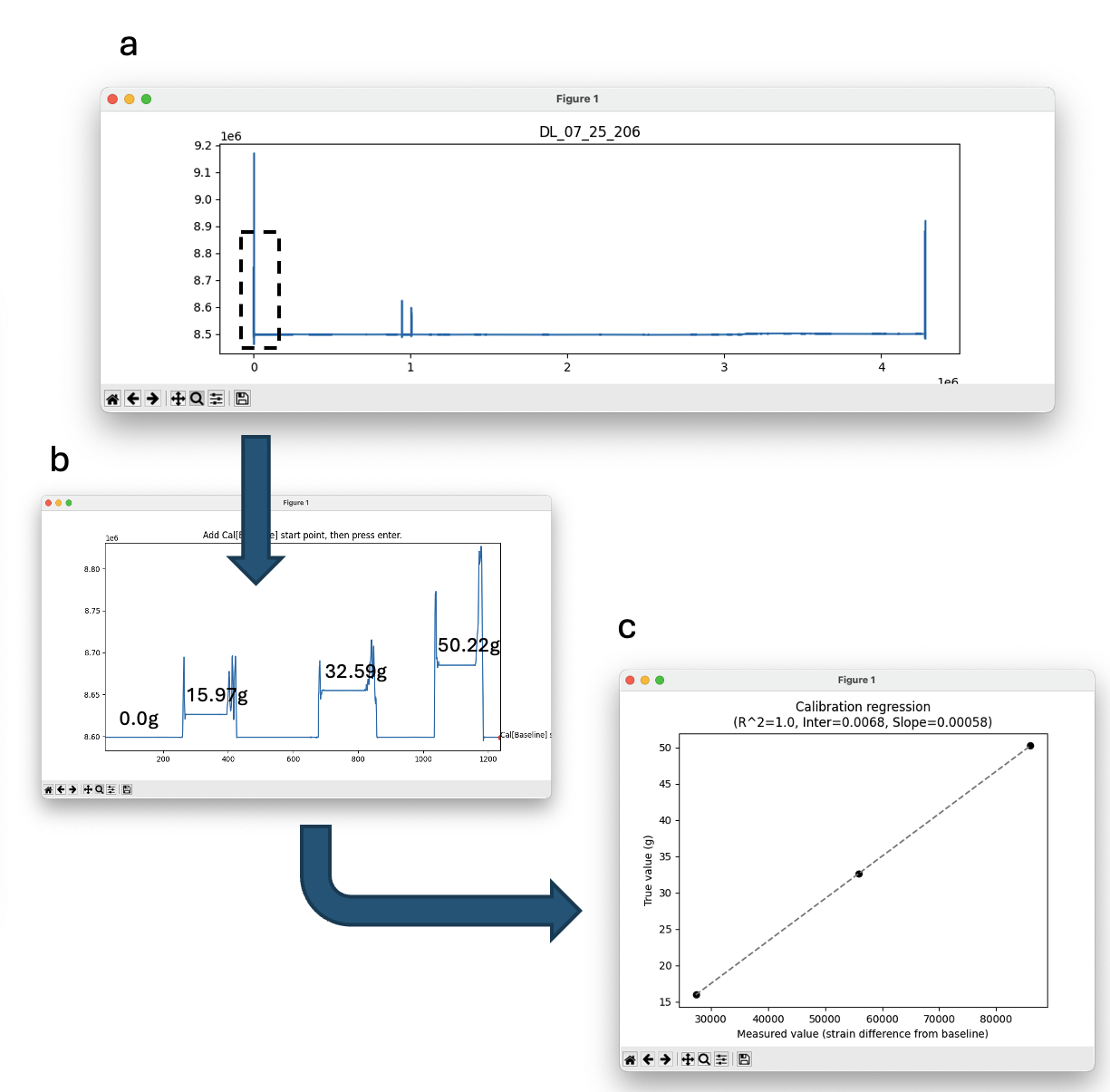


Figure S.4. Calibration procedure. The user isolates the initial calibration from the entire night’s data (a). The calibration procedure can be seen in which there is an initial baseline followed by the placement of 3 known weights on the Weigh Platfform (b). After confirming the choice of the baseline and known weights, a regression result is shown to the user (c). The resulting slope and intercept are used to guage subsequent instances where a bird walks through the BSM. Two of these can be seen approximately 25% through the recording period, as is the backup calibration at the end of the recording period (b).

S.5 - Bird trace - Burrow #385 on 6/20/2024


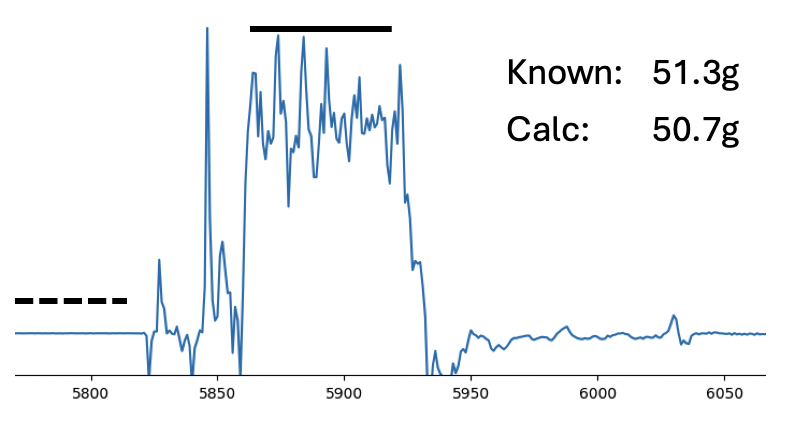


Figure S.5. An example of a trace in which a bird entered the burrow during the validation trials. The dotted line indicates the section of the raw data used for the baseline against which to measure the bird weight. The solid line indicates the section of the trace chosen by the user during the manual procedure. This choice resulted in an estimate that was 0.6g less than the known weight of the bird.

Figure S.6


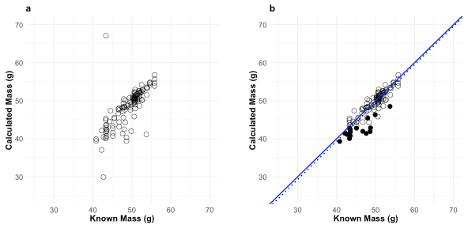


Figure S.6. Validation Results. Birds weighed with an Ohaus scale (Known Mass), then as they passed through the BSM (Calculated Mass). Raw data (a) before filtering (middle 99.8th percentile of historical adult masses) and (b) after manually recalculating values that fell in the lower 10^th^ percentile of historical adult masses. Closed circles represent points after manual recalculation. Dotted line represents the regression of Known Mass on Calculated Mass (F = 450.5, df = 1, 114, r^2^= 0.80). The solid line represents hypothetical perfect correlation.

Figure S.7


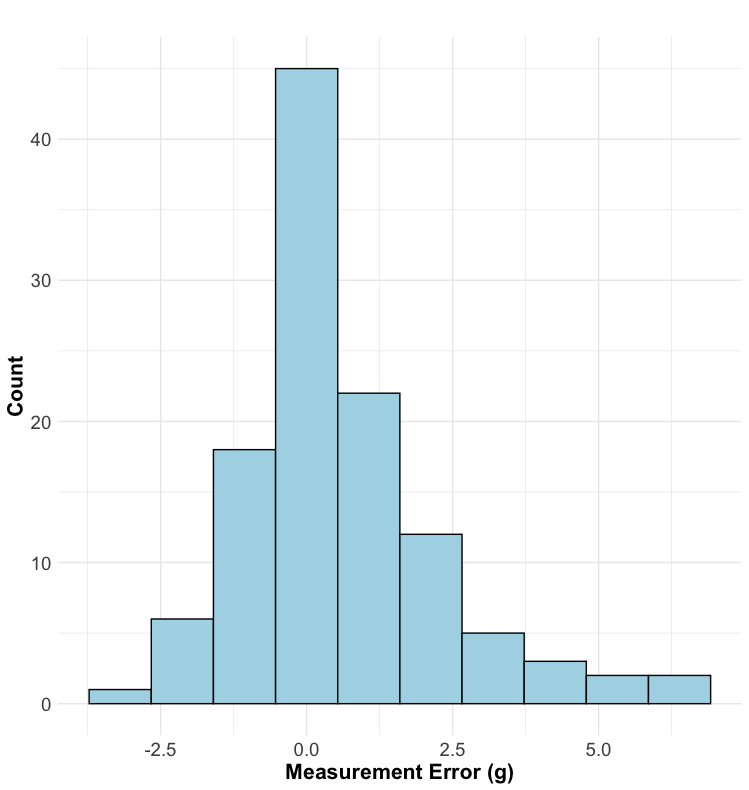


Figure S.7. Validation measurement error (Known Mass – Calculated Mass) for 116 instances of a bird passing through a BSM.

Figure S.8 - Simulation Logic


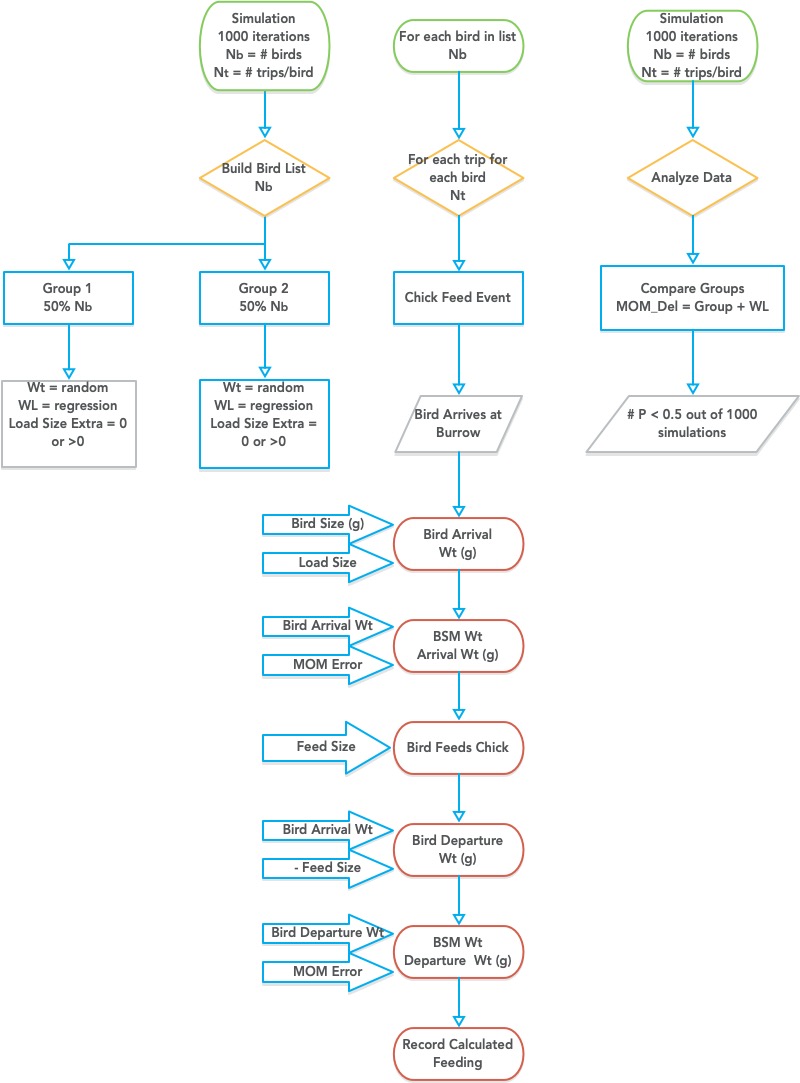


Figure S.8. Simulation Logic. The left column represents the initial setup of the simulation. The middle column describes the details of each feeding event for each trip made by each bird in the simulation. The right column summarizes how the simulation summarizes how the differences between the two groups are assessed.

Figure S.9 - Power Analysis


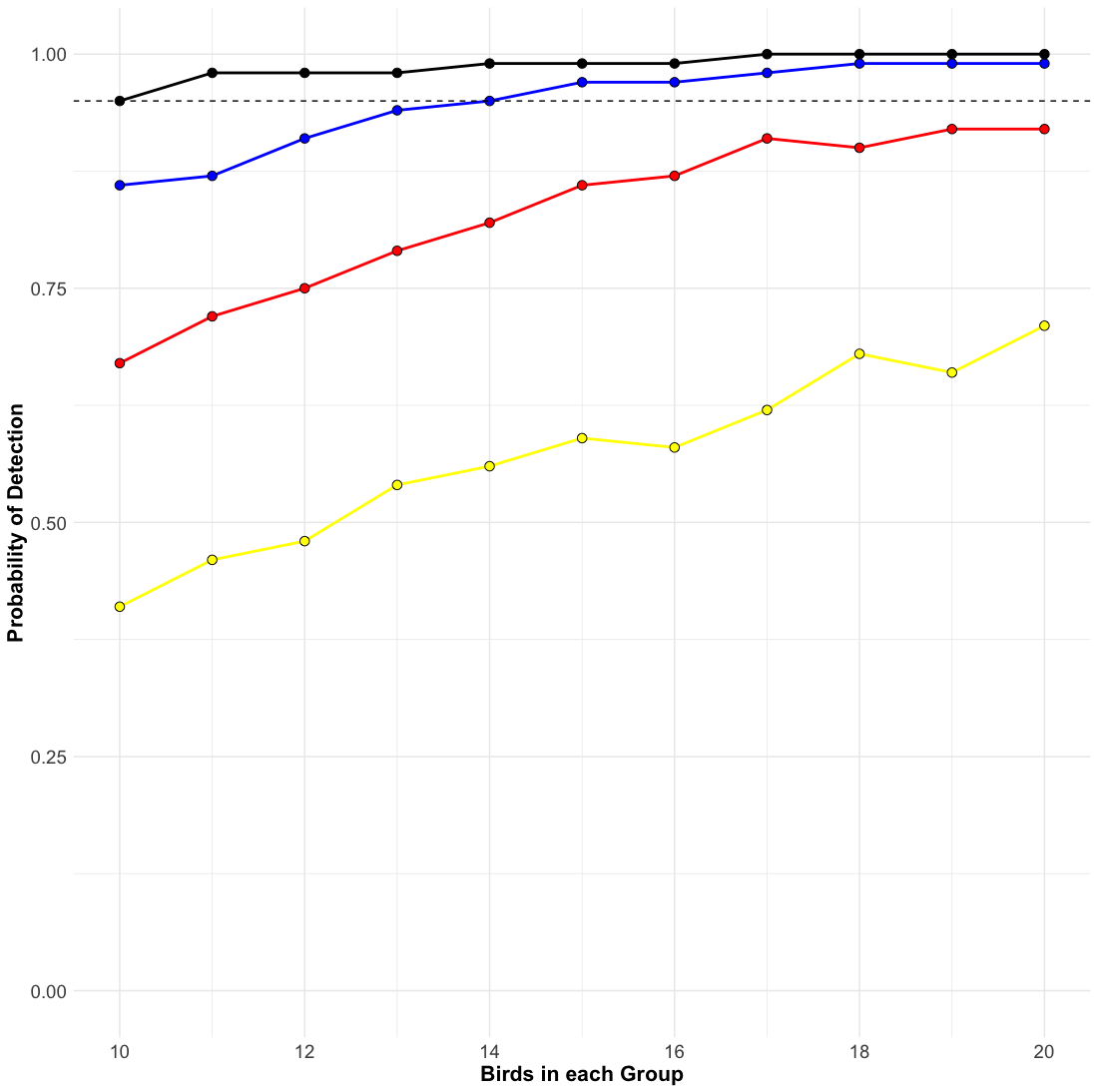


Figure S.9. Power Analysis. Probability of Detection (the proportion of simulations that detected the difference between groups at alpha = 0.05) as a function of sample size (Birds in each group) grouped by the hypothesized proportional difference between the groups (black = 0.25, blue = 0.20, orange = 0.15, yellow = 0.10). Results reflect 1000 simulations at every combination of group size and hypothesized difference. The simulation assumed 8 feeding trips per individual. The dashed line equals the 0.95 probability of detection.
